# Application of Recombinant Human BMP-2 with Bone Marrow Aspirate Concentrate and Platelet-Rich Fibrin in Titanium Mesh for Vertical Maxillary Defect Reconstruction prior to Implant Placement

**DOI:** 10.1155/2021/6691022

**Published:** 2021-10-07

**Authors:** Kamel Alraei, Jameel Shrqawi, Khawlah Alarusi

**Affiliations:** ^1^Department of Oral and Maxillofacial Surgery, Al Noor Specialist Hospital, Mecca, Saudi Arabia; ^2^Department of Oral and Maxillofacial Surgery, Royal Commission Hospital, Al Jubail, Saudi Arabia; ^3^General Dentist, Mecca, Saudi Arabia

## Abstract

Recombinant human bone morphogenetic protein 2 (rhBMP-2) is an alternative bone substitute for extensive maxillary bone defects which avoids the disadvantages associated with other grafting materials. This report details a case of a 32-year-old female with a severe vertical and horizontal maxillary bony defect that developed after tumor removal. She underwent two unsuccessful regenerative surgeries with an iliac bone graft. Reconstruction of the maxillary defect was planned by offlabel use of rhBMP-2/absorbable collagen sponge (ACS) combined with a bone marrow aspirate concentrate (BMAC) and allograft in a titanium mesh covered in platelet-rich fibrin (PRF). Clinical and radiographic evaluations showed good quality and quantity of bone formation, and she was rehabilitated with dental implants and prosthodontic treatment. Based on this case, the use of rhBMP-2 as a graft material appears encouraging with a satisfying outcome. The present case is aimed at reporting the clinical and radiographic effectiveness of rhBMP-2/ACS in combination with PRF and a titanium mesh for severe maxillary bone defects. Future investigations will be required to ascertain the long-term survival of implants in areas grafted with rhBMP-2.

## 1. Introduction

Reconstruction of bone defects after tumor removal or significant trauma is a common practice in maxillofacial surgery; the augmented bone is utilized to house dental implants with subsequent prosthetic rehabilitation. The goal of such a procedure is to maximize both the aesthetic appearance and function [1]. Reconstruction can be achieved by a variety of techniques and materials for bone grafting, including autografts, allografts, and alloplastic grafts. The choice of technique depends on different factors, including the types and degrees of bone loss, surgical prosthetic planning, and the general condition of the patient [2–4].

An autogenous bone graft is considered the ideal grafting material, with the highest success rate due to its osteogenic and osteoinductive capacity. Although it does not trigger an immune response, it is associated with donor site morbidity, poor efficacy for large bone defects, and prolonged recovery time [5, 6].

Allografts are another suitable alternative material for the reconstruction of severely atrophic maxillary bone, with a lower risk of morbidity and shorter recovery time. However, they are associated with an increased risk of infection and require a longer time for graft incorporation [7, 8].

One promising material that has shown successful bone regeneration in maxillofacial defects is recombinant human bone morphogenetic protein 2 (rhBMP-2), which can stimulate angiogenesis and the migration, proliferation, and differentiation of mesenchymal stem cells into bone-forming cells [9]. rhBMP-2 has been used alone or in combination with other graft types with clinical success [10–12]. rhBMP-2 and the absorbable collagen sponge (rhBMP-2/ACS), when used in combination with an allograft, produce bone similar to an autogenous graft and can even be used in large vertical bone augmentation without the need for harvesting a donor bone. This combination can overcome the drawbacks of an autogenous bone graft [13]. Furthermore, platelet-rich fibrin (PRF) is reported to have a positive effect in soft tissue healing and bone formation.

The present case report is aimed at evaluating the clinical and radiographic effectiveness of rhBMP-2/ACS in combination with PRF and titanium mesh in a case of severe maxillary bone defect.

## 2. Case Presentation

### 2.1. Preoperative Examination

**Figure 1:**
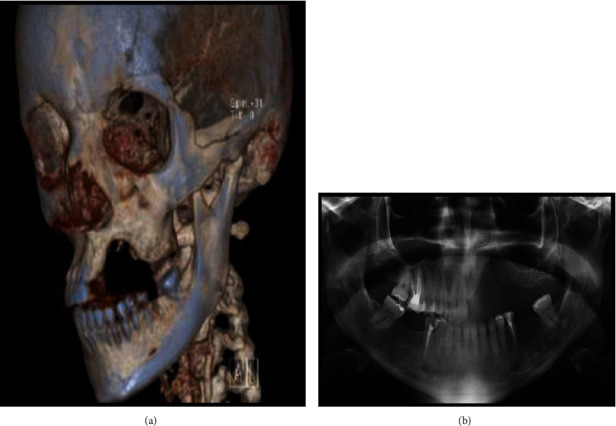
(a) Three-dimensional computed tomography showing partial bony defect of the left maxillary bone. (b) Preoperative panoramic radiography showing a large radiolucent area at the left side of the maxilla.

A 32-year-old female presented to the Oral and Maxillofacial Clinic at Al Noor Specialist Hospital seeking dental implants, with a complaint of absent left upper teeth and jaw, which developed after the removal of a giant cell tumor 1 year previously. The patient had two failed surgeries 1 year previously in which iliac crest bone grafts were used. She was healthy, with no medications, chronic illness, or allergies. Clinical and radiological examinations (3D computed tomography [CT], orthopantomogram [OPG]) revealed a left maxillary bone defect, extending from the left upper central incisor to the left first molar and vertically to the floor of the maxillary sinus (Figure 1). These findings dictated the necessity for bone grafting before the placement of dental implants.

Reconstruction of the maxillary defect was planned with rhBMP-2 combined with bone marrow aspirate (BMA) concentrate (BMAC) and allograft. Titanium mesh was to be used as a carrier and to protect the bone graft component. The mesh was prepared on a study cast to estimate the required size and shape and was sent for sterilization. To overcome the negative impact of the mesh on the oral mucosa, we decided to cover the mesh with PRF. The treatment plan was discussed with the patient, and informed surgical consent was obtained.

### 2.2. Surgery

**Figure 2:**
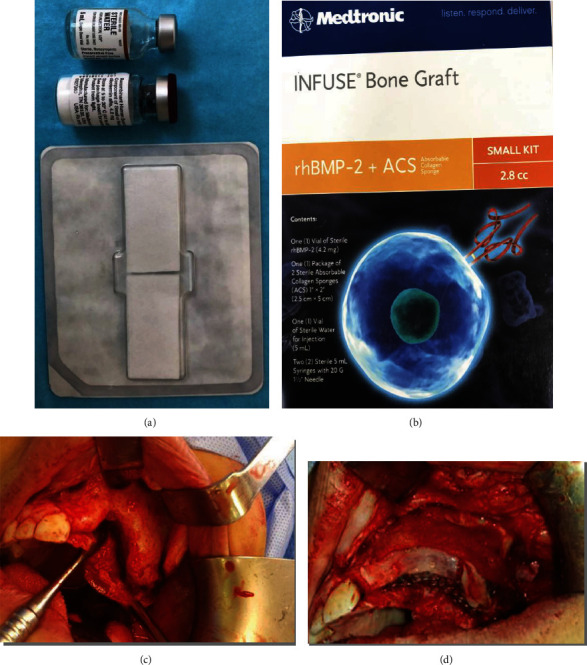
(a) Bone marrow aspirate concentrate. (b) Recombinant human bone morphogenetic protein 2 (rhBMP-2). (c) Maxillary extraosseous vertical ridge defect. (d) rhBMP-2, allograft, and bone marrow aspirate concentrate condensed inside prepared mesh covered by the platelet-rich fibrin.

Under general anesthesia, the patient was placed in the prone position after routine draping and scrubbing of the posterior iliac crest. We aspirated 600 cc of BMA, which was collected into a blood donation bag containing the anticoagulant sodium citrate. The BMA was centrifuged and BMAC was prepared by separating the plasma from the blood (Figure 2(a)).

The patient was then moved to the supine position. After administration of local anesthesia (xylocaine) with 2% adrenaline, an incision was performed 4 mm from the crest towards the palatal side along the maxillary defect, with two releasing incisions vertically extended to the maxillary sulcus. A mucoperiosteal flap was elevated to expose the defect. The recipient area was perforated using a small round burr to increase blood perfusion to the grafting area.

**Figure 3:**
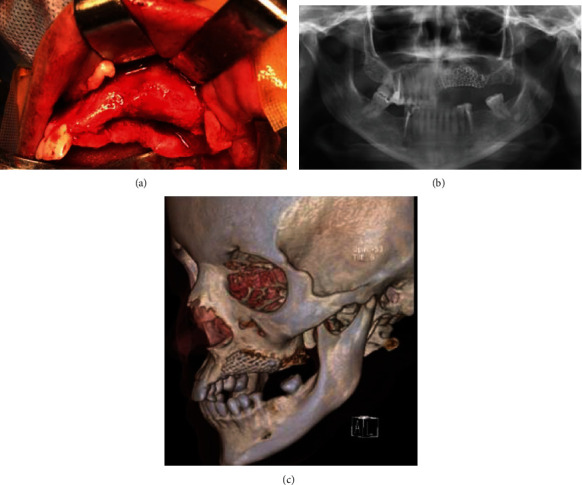
(a) Photographs showing the postoperative view of the maxillary defect. (b, c) Postoperative computed tomography and radiographic evaluation.

rhBMP-2 (Infuse Bone Graft, 8 cc; Medtronic, Minneapolis, MN) was prepared according to the manufacturer's recommendations (Figure 2(b)). ACS was cut into small pieces and mixed with 15 cc of freeze-dried cancellous allograft and BMAC. All these materials were gently condensed inside the previously prepared mesh. Finally, the mesh was adapted to the recipient area and fixed to the maxillary bone from the buccal side with two screws (Figures 2(c) and 2(d)). To protect the thin mucosa from being torn by the mesh, PRF was prepared by collecting four 6 mL tubes of blood. Blood was centrifuged at 3000 rpm for 12 min and condensed in a PRF condenser kit. A PRF membrane was placed over the mesh (Figure 3). The mucoperiosteal flap at the periosteum was scored in advance to achieve tension-free closure.

The patient was administered 1200 mg augmentin (intravenous [IV], 8 h/day for 5 days), 1000 mg paracetamol (IV, 8 h/day for 5 days), and 8 mg dexamethasone (IV, 8 h/day for 3 days).

**Figure 4:**
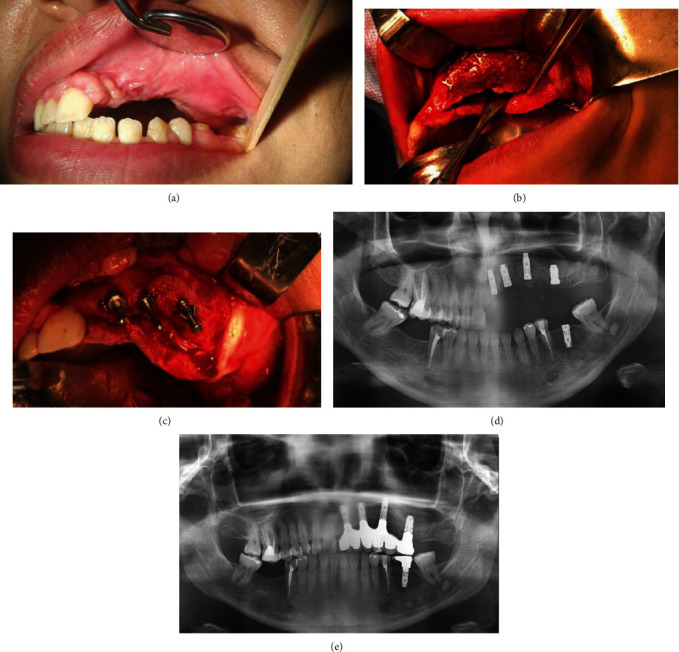
(a) Six months postoperation. (b) Exposing and removing the mesh. (c) Implant placement. (d) Radiograph of bone graft at 8-month follow-up. Note: the significant increase in bone augmentation of the maxilla and implant rehabilitation. (e) Complete regeneration in 3 years following reconstruction of the maxillary defect.

After 5 days, the patient was discharged with weekly follow-up for the first month, then monthly follow-up for 6 months. There was no wound dehiscence or mesh exposure during this time (Figure 4(a)).

### 2.3. Implant Placement, Abutment Connection, and Bridge Delivery

An OPG and CT scan was performed 6 months later to evaluate bone formation. CT revealed a good quantity of bone formation for the accommodation of four implants.

The mesh was exposed and removed under general anesthesia using the same incision. Using a two-stage protocol, four implants (Biomet 3; Palm Beach Gardens, FL) were placed in the left maxilla (3.25 mm × 10 mm, 4 mm × 10 mm, 5 mm × 8.5 mm, 5 × 8.5 mm) and one implant (4 mm × 10 mm) in the left mandible (Figures 4(b) and 4(c)).

Five months later, osseointegration was successful with 35 N/cm of torque, and the prosthetic part was completed.

### 2.4. Follow-Up

Three years after bone augmentation and implant placement, a satisfactory quantity of bone around the implants was visible in a follow-up X-ray.

The patient reported normal function and was satisfied with her cosmetic appearance (Figures 4(d) and 4(e)).

## 3. Discussion

One of the most significant challenges in oral and maxillofacial surgery is the reconstruction of osseous defects, especially in cases of vertical bone augmentation. This is due to the limited osseous walls surrounding the defect, which negatively affect blood supply, graft stability and soft tissue coverage, and the lack of osteoprogenitor cells [14].

For regenerative surgeries, many graft materials have been developed with variable properties. One relatively new material is rhBMP-2, a member of the transforming growth factor-*β* superfamily, which plays an important role in the modulation and differentiation of mesenchymal cells into osteoblasts [15, 16].

rhBMP-2 is the most investigated growth factor for oral and maxillofacial applications that has shown a high success rate in bone defect reconstruction [17]. In 2008, the US Food and Drug Administration approved the clinical use of rhBMP-2 in sinus lifting and localized alveolar ridge defects associated with extraction sockets [16]. Many studies have reported offlabel uses of rhBMP-2 in the reconstruction of maxillary bone defects [14, 16, 18]. Moreover, it has become an alternative to autogenous bone and other bone grafts, as it can overcome the complications associated with harvesting bone from the iliac crest or other donor sites [19]. Furthermore, the new bone induced by rhBMP-2/ACS was found to be similar to autogenous bone and demonstrated good osseointegration [13].

However, rhBMP-2/ACS does not provide enough space for bone formation, especially in vertical and lateral alveolar ridge defects, due to soft tissue compression. Thus, a titanium mesh has become an interesting alternative material [12]. Its first use in oral surgery for bone augmentation was reported by Boyne et al. in 1985 [20]. The advantages of using a titanium mesh with rhBMP-2/ACS are that it can support the soft tissue and prevent it from collapsing into the regenerative site, permitting adequate bone formation. Furthermore, it acts as a scaffold to hold the graft in place [21–23].

However, a titanium mesh has some disadvantages. A second surgery is needed to remove it, and it is associated with a high rate of infection [24]. To prevent wound dehiscence in the present case, in addition to decreasing wound closure tension by adequate scoring of the periosteum, the PRF and collagen membrane was added to cover the titanium mesh as a soft layer between the mesh and oral mucosa to counteract the harmful effects of the mesh on the oral mucosa. Growth factors released from platelets also play a role in healing by attracting undifferentiated stem cells to the site of injury. In addition, PRF plays a role in osteogenesis and angiogenesis [25]. PRF enables a slower release of growth factors leading to better healing [26]. Ozdemir et al. concluded that the use of PRF in conjunction with a titanium mesh increased the quality and quantity of newly formed bone [27].

The case described in this study shows that it is possible to achieve successful augmentation of a maxillary defect and rehabilitation with dental implants by using rhBMP-2 in combination with allografts in a titanium mesh covered by PRF, leading to both graft osteoinduction and osteoconductivity. Additional research is necessary to determine the ideal combination of grafting materials and to ascertain the long-term survival of implants in areas grafted with rhBMP-2.
